# Aerodynamic Evaluation of Flapping Wings with Leading-Edge Twisting

**DOI:** 10.3390/biomimetics8020134

**Published:** 2023-03-24

**Authors:** Lung-Jieh Yang, Vivek Jabaraj Joseph, Yuan-Lung Lo, Wen-Tzu Tang, Balasubramanian Esakki, Saravana Kompala, Paritala Veeranjaneyulu

**Affiliations:** 1 Department of Mechanical and Electro-Mechanical Engineering, Tamkang University, Tamsui, New Taipei City 25137, Taiwan; ljyang@mail.tku.edu.tw (L.-J.Y.); kompalasaravana@gmail.com (S.K.); veeru3nity@gmail.com (P.V.); 2 Department of Civil Engineering, National Taipei University of Technology, Taipei 10608, Taiwan; yllo@ntut.edu.tw; 3 Graduate Institute of Athletics and Coaching Science, National Taiwan Sport University, Taoyuan City 33301, Taiwan; wentzutang@gmail.com; 4 Department of Mechanical Engineering, Vel Tech Rangarajan Dr Sagunthala R & D Institute of Science and Technology, Chennai 600062, India; balasubramaniane@veltech.edu.in

**Keywords:** flapping mechanism, flapping wing micro aerial vehicle (FWMAVs), bevel gears, leading-edge twisting, 3D printing, Kwon3D, MATLAB

## Abstract

The purpose of the current study is to emphasize the characteristics and phenomena of leading-edge twisting in flapping wing vehicles. A fused deposition modeling (FDM) 3D printing method is applied to develop the flapping mechanisms with bevel gears to achieve the leading-edge twisting. Three flapping mechanisms were developed, including simple flapping only (type-A1: normal servo mechanism), flapping with continuous leading-edge twisting (type-B: servo-bevel gear mechanism), and flapping with restricted leading-edge twisting via mechanical stoppers (type-B1: servo-bevel gear mechanism with adjustable mechanical stoppers). Utilizing a low-speed wind tunnel, the aerodynamic performances of these mechanisms are examined by extracting their lift and net thrust forces. The wind tunnel testing data showed that the flapping with restricted leading-edge twisting via mechanical stoppers (type-B1) showed better performance than the simple flapping (type-A1) by 32.9%, and also better performance than the flapping with continuous leading-edge twisting (type-B) by 64%. Next, MATLAB software was used to create the 3D wing surfaces from the instantaneous stereophotography Kwon3D trajectories to fully sketch the leading-edge twisting features. The 2D airfoil cut sections at the mean aerodynamic chord at different stroke moments depict the instantaneous angles of attack to justify the aforementioned wind tunnel testing data and it was verified using a theoretical trajectory model. This comprehensive study using the 3D-printed mechanisms is well suited for the quantitative evaluation of the lift contribution from leading-edge twisting.

## 1. Introduction

The flapping stroke of many birds and insects has two kinematics phases: translational phase and rotational phase [1]. The translational motion, which has upstroke and downstroke, usually keeps less variant angle of attack (AOA) and the leading-edge vortex (LEV) to produce flapping lift [2]. The rotational motion includes pronation and supination. Pronation is the rotation of the wing at the end of the upstroke and supination is the rotation of the wing at the end of the downstroke. Dickinson et al. found that several lift peaks in his *Drosophila melanogaster* scaled model experiment were due to the wing rotation effect, including the rotational circulation and the wake capture, up to a 35% increment in lift [3,4,5].

Dickinson et al. [5] moreover categorized wing rotation as advanced, symmetric, and delayed types. Advanced and symmetric wing rotation occurs before and at the stroke reversal. Both advanced and symmetric wing rotations are beneficial to the flapping lift enhancement. However, delayed wing rotation that happens at some point after the stroke reversal and is bad for the flapping lift [6,7,8,9]. One example of delayed wing rotation is the Golden-Snitch with an oblique figure-of-eight motion at the wing tip [10,11,12,13].

By using a four-bar linkage (FBL) mechanism, few researchers were able to attain a large stroke angle of around 74–170° using a piezoelectric PZN-PT unimorph actuator as well as different wing morphologies, and the flapping frequencies varied around 20–170 Hz [14,15,16,17,18]. Other researchers used FBL mechanisms with more stroke angles, around 74–160°, and their flapping frequency ranged from 0.278 to 15 Hz [19,20,21,22,23]. These prior studies of flapping wing micro air vehicles (FWMAVs) using FBL flapping mechanisms showed their leading-edge twisting or figure-of-eight due to the fluid–structure interaction (FSI) effect by adopting flexible wing frames.

Other researchers applied crank mechanisms and realized a stroke angle in the range of around 48–180° and with a flapping frequency ranging from 8.8 to 60 Hz [24,25,26,27,28,29,30,31]. These mechanisms had more robust translational motion without active leading-edge twisting.

Three scholars have applied active leading-edge twisting in flapping mechanisms using six bar linkage and parallel crank-rockers to augment the lift performance [32,33,34]. Other prior works [35,36,37,38,39,40] also inspire the authors to keep developing new flapping mechanisms.

Previously, the authors have carried out preliminary research on the leading-edge twisting mechanism by using two pairs of servo motors for performing flapping and leading-edge twisting simultaneously [13,41]. The authors moreover adopted bevel gears [8] engaged in the simple flapping mechanism to create the leading-edge twisting synchronously. The bevel gears also helped to reduce the total weight of flapping mechanisms by reducing the number of servo motors from four to two. The leading-edge carbon spars were attached to the bevel gear’s rotational axis to achieve the wing twisting continuously.

A wind tunnel with a six-axis load cell to estimate the aerodynamic performance of flapping mechanisms is always necessary in general [6,9,11,12]. Another important method used in this study is via visual sensing technology [10]. In general, gait analysis is applied on athletes using visual sensing technology to improve their efficiency in terms of force and body movements [42]. It requires many markers to highlight the motion path of the subject [43]. This technique led the authors to find out the flapping wing flying pattern in Golden-Snitch FWMAV by using nine light-emitting diode (LED) markers. The LED trajectories were seen through high-speed cameras during flapping, using coordinate transformation by Kwon3D software [44,45]. The oblique figure-of-eight trajectory of the FWMAV Golden-Snitch was confirmed experimentally through this method. The detailed 3D coordinates, as well as the 2D cross sections of the deforming wing profiles corresponding to different time steps, were obtained from the experiment, which unveils the shape of the wing profile. In order to analyze the real kinematics of the wing for different leading-edge twisting mechanism designs, this method was applied herein.

So, the following two parts of the work will be demonstrated in this paper: (1) the wind tunnel testing is carried out to find the aerodynamic forces for different flapping mechanisms with or without leading-edge twisting; (2) the visual motion sensing using Kwon3D software for the same flapping mechanisms in (1) to find out their individual 3D trajectories, 3D wing surfaces, and 2D cut sections using MATLAB software. The leading-edge twisting mechanism designs will first be demonstrated in the next section.

## 2. Materials and Methods

To study and achieve the leading-edge twisting, the authors surveyed various flapping mechanisms and explained these in the previous section. Henceforth, several flapping mechanisms with leading-edge twisting were designed and developed in this work. To compare the leading-edge twisting flapping mechanism, authors developed a normal servo mechanism (type-A1) which has no leading-edge twisting but just translational flapping. The flapping mechanisms with different configurations are briefly explained below and the aerodynamic force measurements were carried out and discussed. Aided by KwonCC, Kwon3D software, MATLAB 3D surfaces, MATLAB 2D cut sections, and a theoretical model of wing kinematics, the force signals were analyzed and discussed.

### 2.1. Type-A: All Servo Mechanism

**Figure 1:**
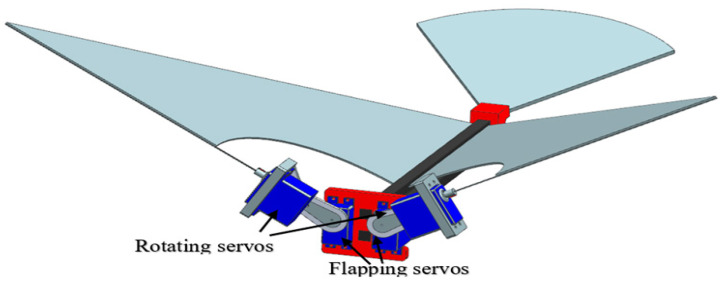
All servo flapping mechanism [6].

The idea to try out the first-generation flapping mechanism with leading-edge twisting is shown in Figure 1 [6]. This mechanism consists of four servo motors, two for translational kinematics and two for rotational kinematics. The leading-edge twisting servo motors actuate only at the stroke reversal. The benefit of using servo motors in the flapping mechanism is the high power-to-torque ratio during translational and leading-edge twisting motions. A 3D printing technique was used to fabricate the less heavy parts and carbon rods were applied to the fuselage as well as at the wings to function like spars.

Generally, the FWMAVs use mechanical gears and DC motors for flapping, this servo-based ornithopter in Figure 1 uses pulse width modulation (PWM) signals and an Arduino microcontroller to actuate. Due to the constraints of weight reduction and less power consumption, new flapping mechanisms are required.

### 2.2. Type-A1: Normal Servo Mechanism

**Figure 2:**
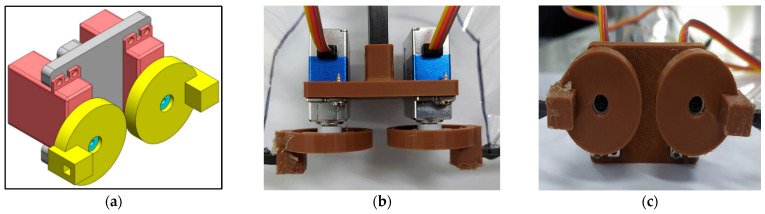
Type-A1 normal servo mechanism: (**a**) CAD model; (**b**) top view of 3D-printed mechanism; (**c**) front view of 3D-printed mechanism [6,9,15].

**Figure 3:**
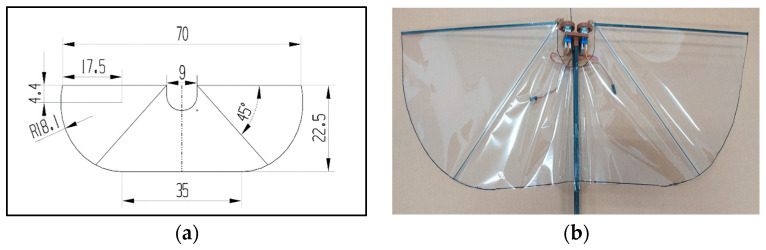
Wing geometry: (**a**) CAD model; (**b**) fuselage attached with PET wing [6].

This type-A1 flapping mechanism consists of 2 servo motors for translational flapping motion without leading-edge twisting. CAD model, 3D-printed parts, and the assembled mechanism are shown in Figure 2. The wing material assigned in this whole study was polyethylene terephthalate (PET). The wing has span of 70 cm, a chord length of 22.5 cm, and a semi-elliptical wing area of 1406.8 cm^2^ as shown in Figure 3. This mechanism has a zero-phase lag between the 2 servo motors actuated with PWM signals. Arduino microcontroller is used to give the digital signal input to the servos.

### 2.3. Type-B: Servo-Bevel Gear Mechanism

**Figure 4:**
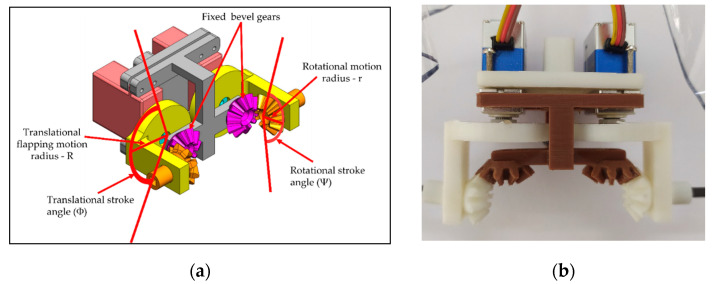
Type-B bevel gear mechanism: (**a**) CAD model; (**b**) top view of 3D-printed mechanism [6,9,15].

This type-B flapping mechanism consists of one pair of servo motors for translational motion and one pair of bevel gears to attain the leading-edge twisting at the same time. The bevel gear rotation is entirely reliant on the gear ratio as well as the translational stroke angle produced by servo motors as shown in Figure 4.

To maintain the same conditions as previously mentioned, the wing design of type-B was similar to type-A1 flapping mechanism except for the bevel gears. The pink-colored bevel gear in Figure 4a is the fixed gear and the orange color bevel gear is rotating gear. The kinematic relation of the bevel gear motion is described below.

R·Φ = r·Ψ (the same arc distance)(1)

The quantity R·Φ in Equation (1) represents the product of the disc radius R and stroke angle Φ for the translational motion of the flapping wing spar; the quantity r·Ψ represents the product of the bevel gear radius r and leading-edge twisting angle Ψ for the rotational motion of the followed wing spar twisting. This condition is always satisfied for the continuous leading-edge twisting, and it is evident from the type-B flapping mechanism that the stroke angle Φ is directly proportional to leading-edge twisting angle Ψ. Through the bevel gear engagement, the rotating arc distances for the two gears are the same and Figure 4a demonstrates the relationship between each variable in Equation (1) to understand the leading-edge twisting in a better way.

### 2.4. Type-B1: Servo-Bevel Gear Mechanism with Adjustable Mechanical Stopper

**Figure 5:**
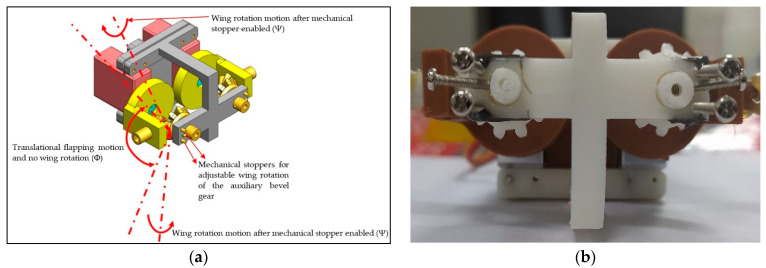
Type-B1 mechanism with mechanical stoppers: (**a**) CAD model; (**b**) front view of 3D-printed mechanism [6,9,15]; the duty percentage for rotation is 74.7%; it produces no leading-edge twisting when the pin screw freely travels between the two stoppers (24.3% of the total cycle) and the auxiliary bevel gear rotates with the pin screw to have no leading-edge twisting.

A coherent way to modify the type-B mechanism is to improve the net thrust. It was suspected that the continuous leading-edge twisting throughout the flapping in type-B mechanism is power-consumptive as well as unwanted. So, it was necessary to fine tune the leading-edge twisting at the appropriate and precise timing. Hence, the mechanical switchable stopper is added to the bevel gear to control it, as shown in Figure 5, and it is named the type-B1 mechanism.

Type-B1 mechanism is similar to type-B mechanism except for changing the fixed bevel gear in Figure 4 to the auxiliary bevel gear in Figure 5. The mechanical stoppers control when the auxiliary bevel gears become fixed bevel gears and then turn on the leading-edge twisting temporarily. Hence, the whole motion is not a continuous but a selective leading-edge twisting. The adjustable positions of mechanical stoppers determine when to engage the leading-edge twisting before stroke reversals and also ensure the advanced leading-edge twisting to benefit the flapping lift. The wing parameters of type-B1 in Figure 5 are the same as type-B in Figure 4 to avoid mismatching comparison.

### 2.5. Testing Methodology

The testing of flapping wing was carried out in two methods: (**a**) aerodynamic fore measurement using a wind tunnel and (**b**) wing profile analysis using visual motion sensing technique.

- Wind tunnel testing

**Figure 6:**
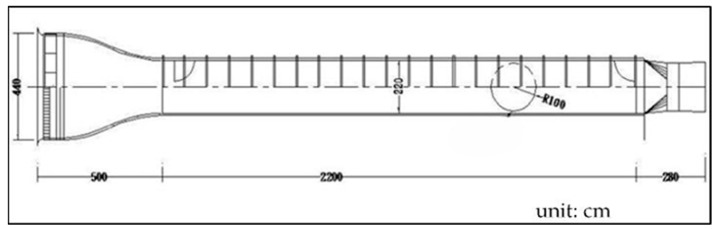
Dimensions of the large wind tunnel.

**Figure 7:**
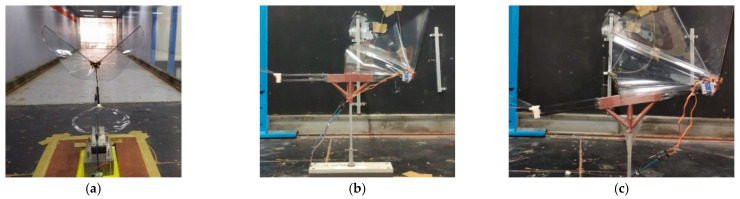
MAV mounted on force gauge inside the wind tunnel: (**a**) front view; (**b**) side view of 0° inclined angle; (**c**) side view of 15° inclined angle.

The classical lift and net thrust signals are shown in [9] as a reference to this work. They were obtained from Golden-Snitch FWMAV of 20 cm wingspan for the wind speed of 3 m/s and 3.7 V driving voltage [10,11,13,14,41,46,47]. However, the flapping wing in Figure 3 is of a 70 cm wingspan, much larger than the Golden-Snitch. Therefore, after the assembly of all three flapping mechanisms and the wing in Figure 3, the aerodynamic force measurement was performed in a larger wind tunnel (Figure 6) in the Department of Civil Engineering of Tamkang University with the testing section specification as 15 m (length) × 2.2 m (width) × 1.8 m (height). Table 1 shows the details of wind tunnel. The wind speed range herein is only 1.5–4 m/s. Figure 7a shows how the FWMAV was mounted on the force gauge inside the wind tunnel.

The inclined angles of the FWMAV in Figure 7b,c ranged from 10° to 35° and also varied with driving voltages of 1.25 V with a flapping frequency of 1.5 Hz, 2.5 V with a flapping frequency of 2.0 Hz, and 5 V with the corresponding flapping frequency of 2.5. InstruNet World software was used to collect the unsteady data from the six-axis force gauge and the load cell can measure up to 200 gf of lift force and 100 gf of net thrust force. The margin error of 0.2% occurs due to the nonlinearity and hysteresis [15]. The wind tunnel force signals were transferred into MATLAB to eliminate the unnecessary noises. About 10,000 points of raw data and 10 flapping cycles of lift and net thrust (thrust minus drag) were averaged to one data point in type-A1, -B, and -B1 mechanisms according to different flight conditions of inclined angle, driving voltage, and wind speed.

- Kwon3D analysis

As mentioned earlier in the Introduction section about the visual motion sensing, KwonCC and Kwon3D are software through which the trajectories can be found for any mobile object. This technique requires reflective markers which can glow at low light conditions to identify all the markers.

KwonCC requires two calibration videos with different angles captured with an angle interval of 120°. For the self-made calibration frame, 23 control points with given 3D coordinates were assigned to denote the virtual domain, and the calibration frame was scaled to 100 cm^3^ in order to cover all the FWMAV trajectory points in the future without missing. Inside this virtual domain, the object motion test was carried out. Again, two angles with the interval of 120° were selected to capture the calibration frame images through 2 high-speed cameras with 1000 frames per second (fps). Direct linear transformation (DLT) method was used in KwonCC to find the 3D coordinate transformation of the calibration frame. To ensure the points accuracy, the error must be reduced to a very low value of around 0.19–0.3 cm. Three flapping cycles were processed in order to obtain the wing profile shapes at each instance. The 3D coordinates of the control points were extracted and exported for further post processing.

**Figure 8:**
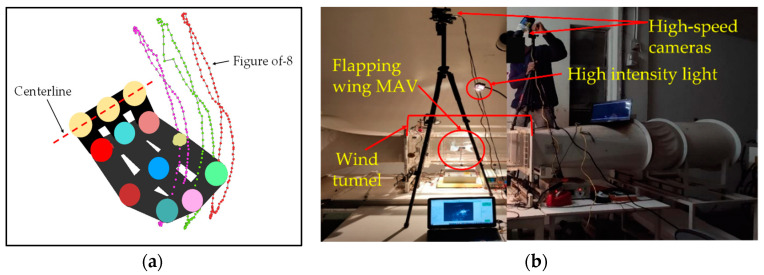
Kwon3D analysis of Evans mechanism: (**a**) wireframe and trajectories of control points; (**b**) experimental setup with two cameras [10].

The flapping wing motion video was then processed using Kwon3D software. To identify the control points on the wing surface, the LEDs were applied over there as the reflective markers which can glow at low light. The wing surfaces had 12 control points, in which the 3 points were assumed to be the centerline and the remaining 9 control points were moving points, as shown in Figure 8a. The slow-motion video was then processed by each frame and the control points were selected precisely. Point positions of each control point were then calculated using the Kwon3D solver. By this method, the control points can be seen as a wireframe and the trajectories of each point at each time frame can be observed. Figure 8b shows the setup of Kwon3D analysis of the first angle and second angle cameras.

The obtained point position data were exported into excel format and imported to MATLAB for post processing and to achieve the 3D wing surfaces. The point position data are a function of coordinates x, y, z, and time t, so the function is f(x, y, z, t) where the changing x, y, z coordinates are a function of time t and the surface was generated. The surface generation through MATLAB is discussed further in the Results and Discussion section.

## 3. Results and Discussion

Three flapping mechanisms, type-A1 servo flapping mechanism, type-B servo with bevel gear flapping mechanism, and type-B1 servo with bevel gear and mechanical stoppers, were designed and fabricated using a 3D printing technique. The aerodynamic force evaluation was carried out for each mechanism. The enormous amount of data was extracted using the wind tunnel for each flapping mechanism for different inclined angles, wind speeds, and driving voltages. Aerodynamic force measurements from the wind tunnel were then averaged and plotted into lift and net thrust. Subsequently, the cruising conditions for all three flapping mechanisms were identified.

### 3.1. Type-A1: Normal Servo Mechanism

**Figure 9:**
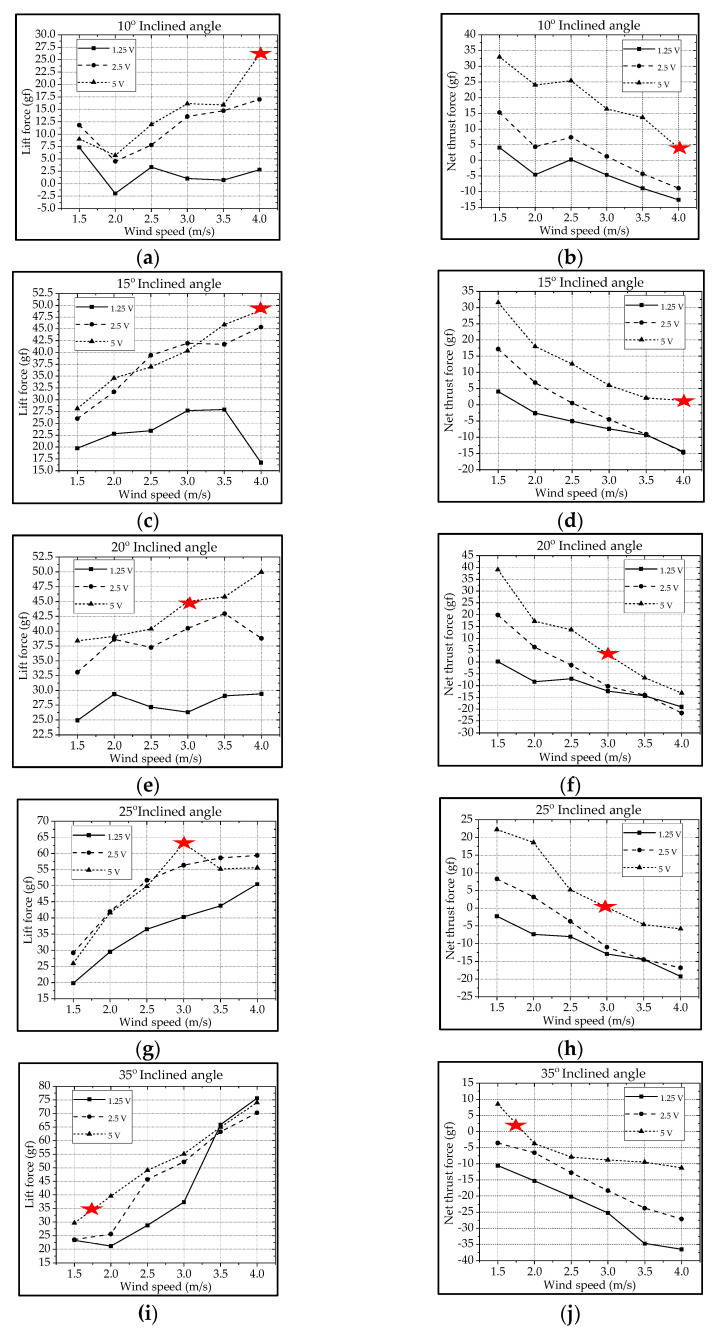
Type-A1 flapping mechanism’s lift and net thrust at different inclined angles: (**a**,**b**) 10°; (**c**,**d**) 15°; (**e**,**f**) 20°; (**g**,**h**) 25°; (**i**,**j**) 35°.

For the type-A1 mechanism, the authors extracted the cruising conditions from Figure 9 and summarized them in Table 2. The best cruising condition with the largest cruising lift of 63.2 gf is at the inclined angle of 25° and the wind speed of 3.0 m/s subject to the driving voltage of 5 V. The lift increases with the increase in driving voltage and wind speed. Parallelly, the net thrust decreases with the increase in wind speed due to the increasing drag. In fact, the inclined angle and wind speed herein were coupled together, and for instance the authors could not keep a constant cruising speed, which is defined as the condition where the net thrust is zero (thrust = drag; marked with 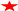) [13,46].

**Figure 10:**
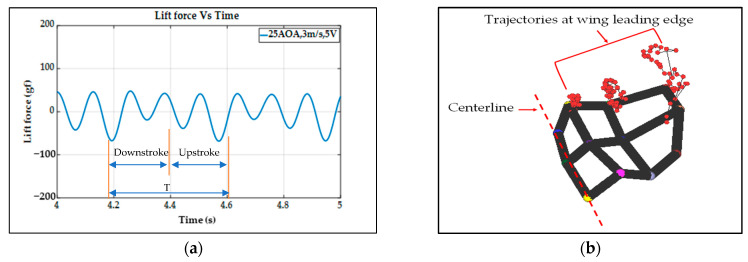
Type−A1 servo flapping mechanism: (**a**) unsteady lift signal; (**b**) wireframe and trajectories of control points.

In Figure 10a, one lift signal is obtained after eliminating the noise using the Fast Fourier Transform (FFT) method with a threshold of 45 Hz in the type-A1 mechanism; the lift signal waveform looks decent, and the aerodynamic performances can be studied in this force signal curve. To verify this lift signal, the visual motion sensing result is hoped to be compared and justified. One 3D trajectory of the nine-LED control points on the wing is shown in Figure 10b.

The type-A1 mechanism had a translational flapping motion, with a total cruising lift of 63.2 gf obtained at the cruising conditions of 25° inclined angle, 5 V driving voltage, and 3 m/s wind speed. The authors used MATLAB software to interpolate the 12-point trajectory of Figure 10b to the 3D wing surfaces of Figure 15a–h to demonstrate the flapping feature. Downstroke motion is shown in Figure 15a–d and the upstroke is shown in Figure 15e–h, which has been linked to the lift signal in Figure 10a and the figure-of-eight wing tip motion in Figure 16a. The 2D airfoil cuts of the 3D surfaces are also taken using MATLAB at the mean aerodynamic chord and are shown in Figure 17a,b. Combining these 2D airfoil cuts and the relative incident velocity at the leading edge [46], it is easy to understand the instantaneous lift change of the simple flapping motion without leading-edge twisting.

On checking the lift peaks in Figure 10a, according to the Dickinson’s three flapping lift mechanisms, i.e., delayed stall, rotational lift, and wake capture [2], there should be no rotational lift in this case. Both the first peak during the downstroke and the center peak during the upstroke may be due to wake capture. The second peak during the downstroke or at the supination is not very clear. By crosschecking 3D profiles in Figure 15a–h and 2D cuts in Figure 17a,b, there is only a simple flapping motion at the inclined angle of 25° and it is supposed to have the delayed stall effect all the way. The induced figure-of-eight wing tip motion in Figure 16a is similar to the fluid–structural interaction (FSI) phenomenon along the streamwise direction of Golden-Snitch in [9]. In Figure 17a, the instantaneous angle of attack (AOA) is shown for the type-A1 mechanism during the downstroke, and it ranges from 61.2° to 67.3° and the maximum lift produced is 63.2 gf. In Figure 17b, during the upstroke the instantaneous AOA ranged from 3.2° to −6.9° and the negative lift forces were canceled when averaging the overall lift data for one full flapping cycle.

### 3.2. Type-B: Bevel Gear Mechanism

**Figure 11:**
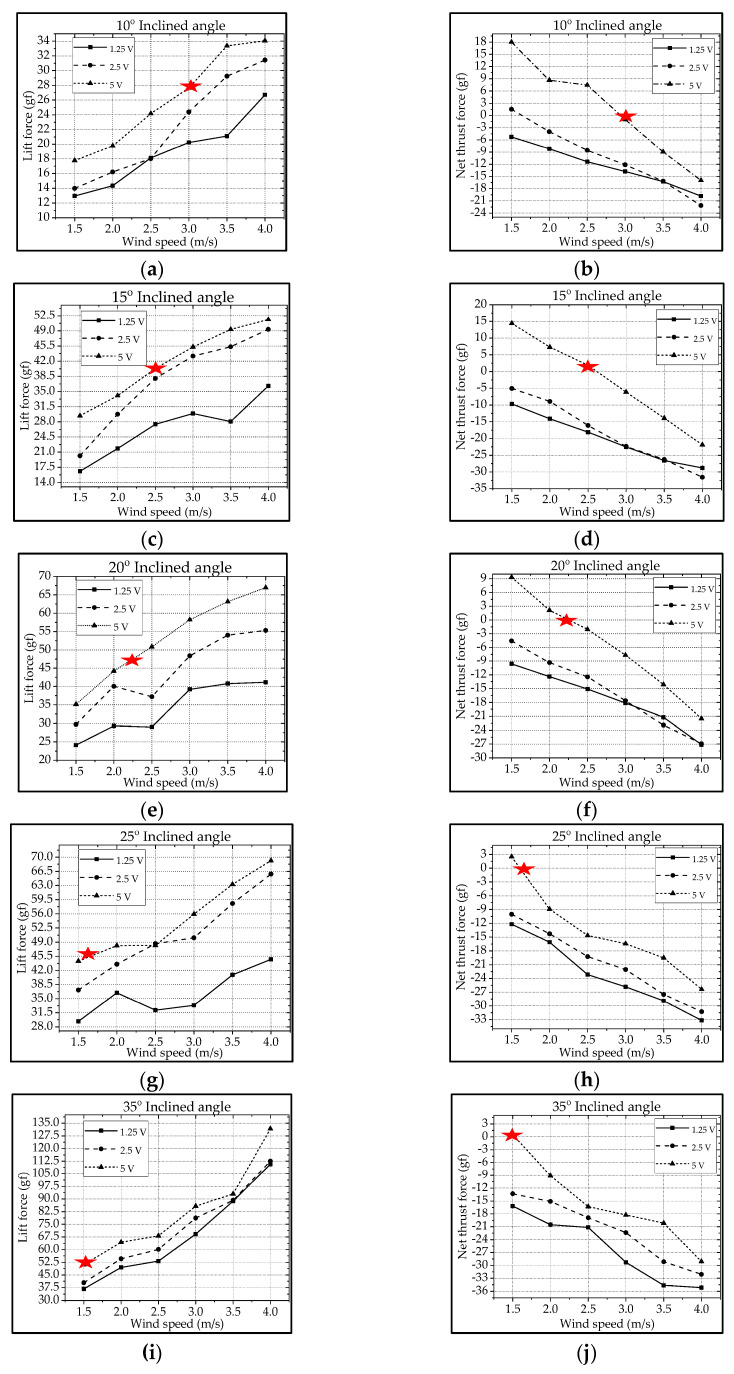
Type−B flapping mechanism’s lift and net thrust at different inclined angles: (**a**,**b**) 10°; (**c**,**d**) 15°; (**e**,**f**) 20°; (**g**,**h**) 25°; (**i**,**j**) 35°.

**Figure 12:**
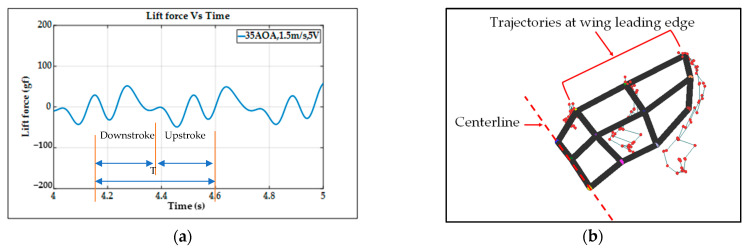
Type−B bevel gear flapping mechanism: (**a**) unsteady lift signal; (**b**) wireframe and trajectories of control points.

Similar to the type-A1 mechanism, the type-B mechanism with its flapping wing after fabrication and assembly was sent to the wind tunnel testing for measuring the aerodynamic forces and for further signal processing. The aerodynamic lift and net thrust were measured for different inclined angles, wind speeds, and driving voltages as shown in Figure 11. The conductive cruising conditions including the cruising speeds and the cruising lift for the type-B mechanism of continuous leading-edge twisting are summarized in Table 3. In Figure 12a, after the noise elimination in the lift signal of the type-B mechanism, more peaks in the lift signal can be seen, and it is supposed to attain more average lift. After the cruising conditions were investigated, it was noted that the cruising lift forces of the type-B mechanism (continuous leading-edge twisting) were worse than the type-A1 mechanism (no leading-edge twisting).

For example, although the lift values in Figure 11a of the type-B mechanism seem higher than the type-A1 mechanism in Figure 9a, the ineffective production of net thrust due to the continuous leading-edge twisting in the type-B mechanism, which is beyond the need herein, may be responsible for the worse result at cruising conditions. In this type-B mechanism, there is a smaller thrust or larger drag during the stroke reversal. A much lower net thrust reduces the cruising speed from 3.0 m/s to 1.5 m/s and results in the smaller cruising lift of 51.1 gf; this means that the continuous leading-edge twisting needs modification. In addition, the 3D trajectory of the nine-LED control points on the wing is shown in Figure 12b.

The type-B mechanism had both the translational flapping and leading-edge twisting, with the total cruising lift of 51.1 gf obtained at the cruising condition of 35° inclined angle, 5V driving voltage, and 1.5 m/s wind speed. The authors used MATLAB software to interpolate the 12-point trajectory of Figure 12b to the 3D wing surfaces of Figure 15i–p to demonstrate the flapping feature. Downstroke motion is shown in Figure 15i–l and the upstroke is shown in Figure 15m–p, which has been linked to the lift signal in Figure 12a and the figure-of-eight wing tip motion in Figure 16b. The 2D airfoil cuts of the 3D surfaces are also taken using MATLAB at the mean aerodynamic chord and shown in Figure 17c,d. Combining these 2D airfoil cuts and the relative incident velocity at the leading edge [46], it is easy to understand the instantaneous lift change of the flapping motion with continuous leading-edge twisting.

On checking the lift peaks in Figure 12a, there should be strong leading-edge twisting motion in this case. If both the first peak during the downstroke and the center peak during the upstroke are still due to wake capture, the second peak during the downstroke or at the supination right now is not obvious. By crosschecking 3D profiles in Figure 15i–p and 2D cuts in Figure 17c,d, the gradual leading-edge twisting is seen all the way during the downstroke and upstroke, and changed the inclined angles hugely at the stroke reversals (both pronation and supination). This kind of inclined angle changing is not a leading-edge twisting, but actually causes negative instantaneous AOA, which is not the same as the inclined angle and is bad for the lift generation herein. In Figure 17c, the instantaneous AOA is shown for the downstroke, and it ranges from 31.5° to 55.1°, and the maximum lift produced is 51.1 gf. In Figure 17d, during the upstroke, the instantaneous AOA varied from −23.1° to −54.8° and the negative lift forces were hugely created when averaging the overall lift data for one full flapping cycle, which resulted in less positive lift. In addition, the figure-of-eight in Figure 16b is much more slender than the case of type-A1 simple flapping. It reveals the weak FSI effect due to the lower thrust force generation and as a consequence results in smaller cruising speed and lift.

### 3.3. Type-B1: Bevel Gear Mechanism with Adjustable Mechanical Stopper

**Figure 13:**
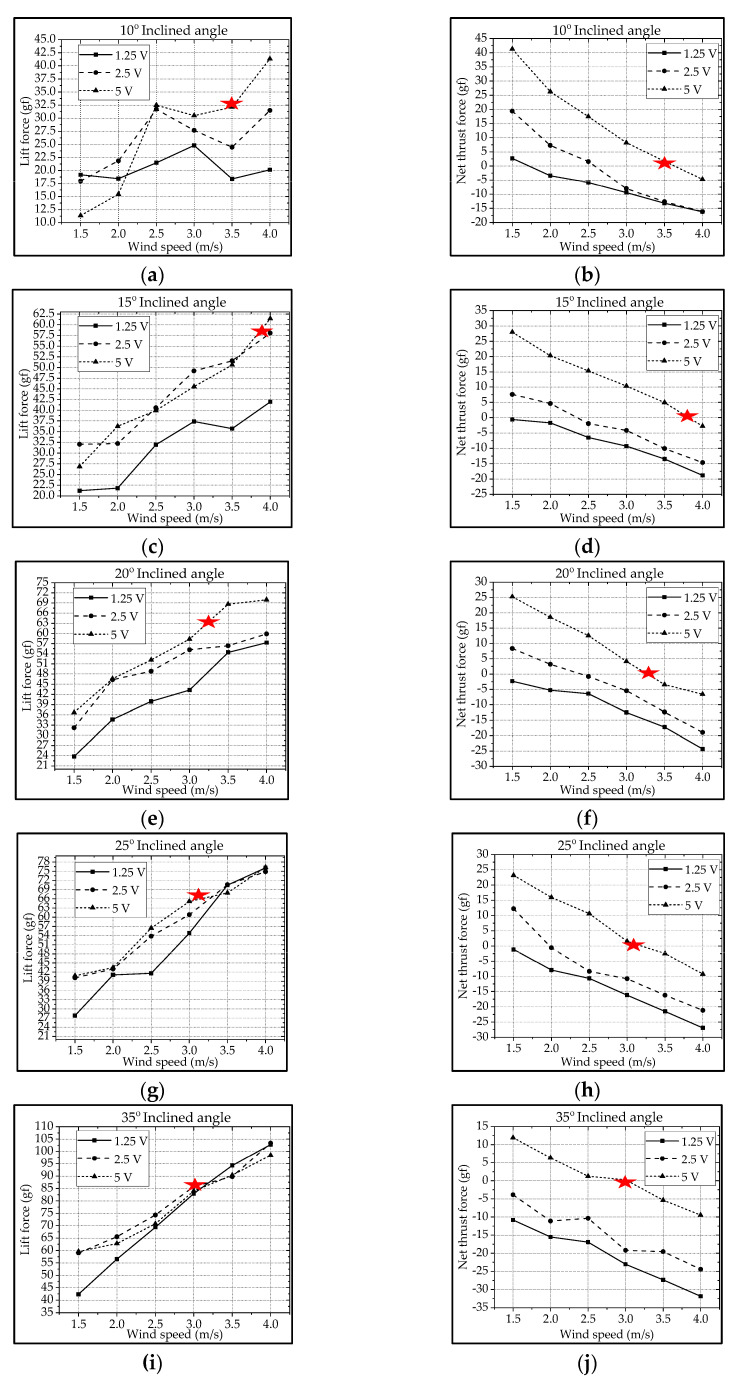
Type-B1 flapping mechanism lift and net thrust at different inclined angles: (**a**,**b**) 10°; (**c**,**d**) 15°; (**e**,**f**) 20°; (**g**,**h**) 25°; (**i**,**j**) 35°.

Similar to the previous treatment of the type-A1 and type-B mechanisms, the type-B1 mechanism with its flapping wing after the fabrication and assembly was sent to the wind tunnel testing for measuring the aerodynamic forces and further signal processing. The aerodynamic lift and net thrust were measured for different inclined angles, wind speeds, and driving voltages shown in Figure 13. The conductive cruising conditions with the cruising speeds and the cruising lift for the type-B1 mechanism with selective leading-edge twisting are also summarized in Table 4.

The maximum lift 84 gf of the type-B1 mechanism happened at a cruising speed of 3 m/s. That is 33% higher compared to the 63.2 gf of the type-A1 mechanism at a cruising speed of 3 m/s and 64% higher than the 51.1 gf of the type-B mechanism at a cruising speed of 1.5 m/s.

**Figure 14:**
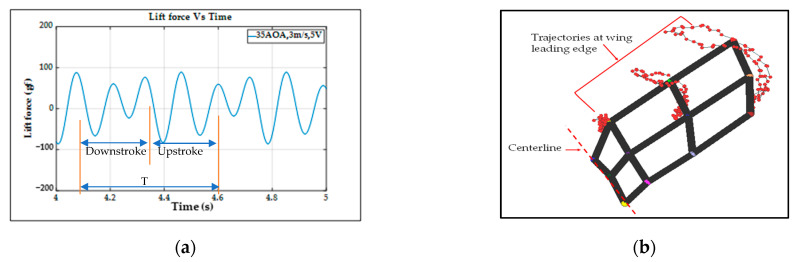
Type-B1 bevel gear with mechanical stopper flapping mechanism: (**a**) unsteady lift signal; (**b**) wireframe and trajectories of control points.

The lift signal of the type-B1 mechanism in Figure 14a also showed the big lift peak before the stroke reversal (i.e., the advanced leading-edge twisting) and induced a big increase in averaged lift value. Again, the 3D trajectory of the nine-LED control points on the wing is shown in Figure 14b. The trajectories will be further processed in MATLAB software to generate a 3D surface to visualize the wing surface for the discussion in Section 3.

**Figure 15:**
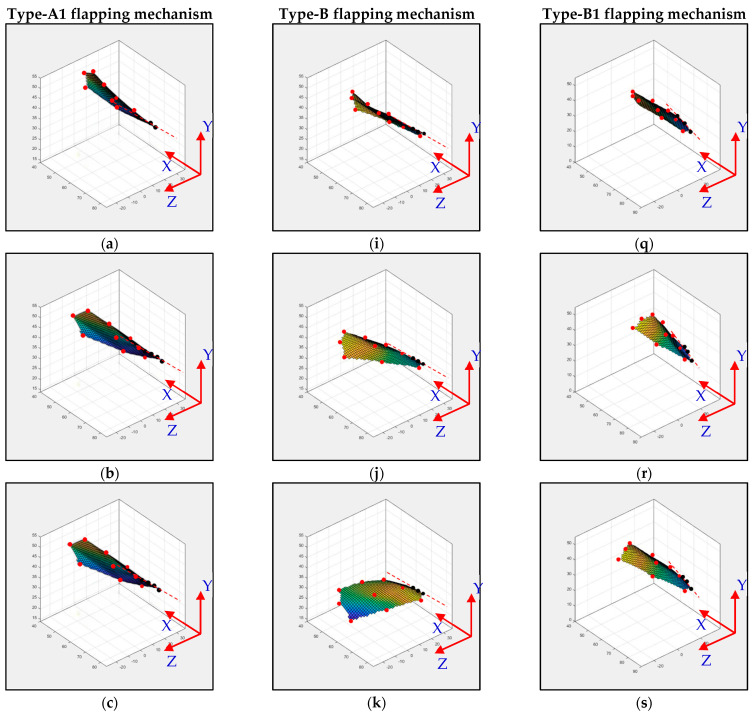

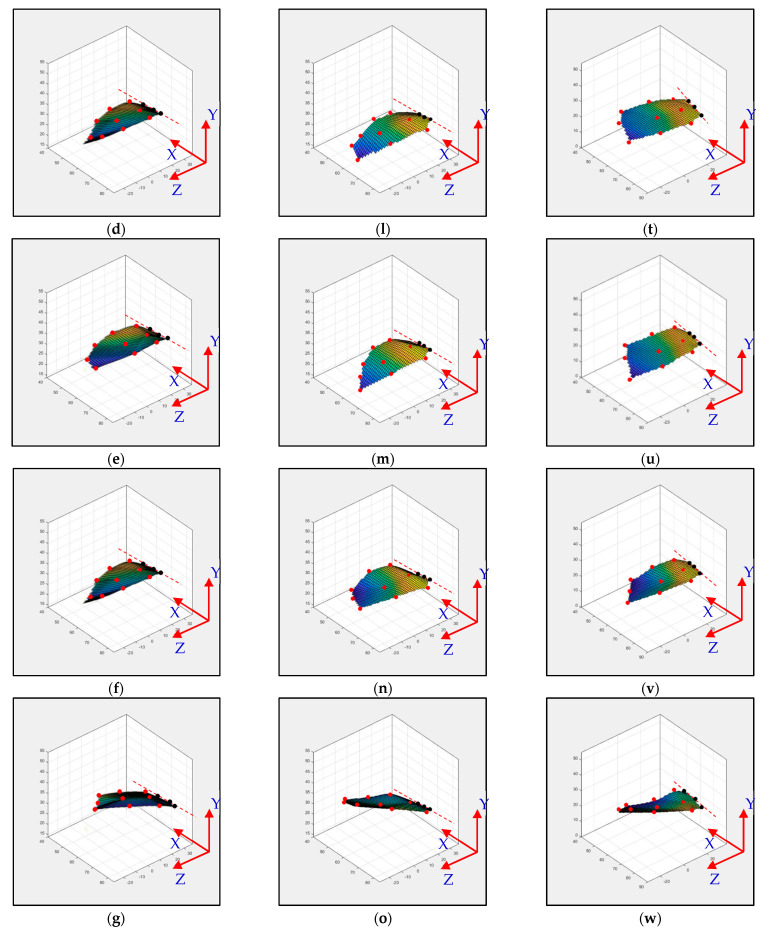

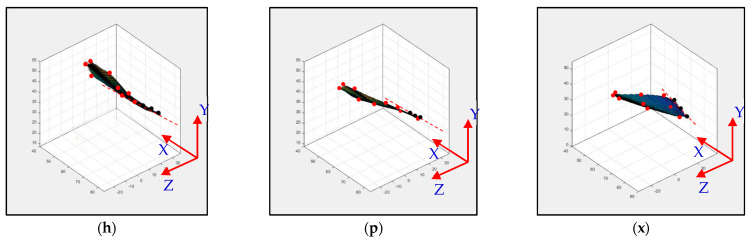
Flapping mechanism’s 3D analysis results: (**a**–**d**) downstroke in type−A1 flapping mechanism wing 3D wing surfaces at different time steps; (**e**–**h**) upstroke in type−A1 flapping mechanism wing 3D wing surfaces at different time steps; (**i**–**l**) downstroke in type−B flapping mechanism wing 3D wing surfaces at different time steps; (**m**–**p**) upstroke in type−B flapping mechanism wing 3D wing surfaces at different time steps; (**q**–**t**) downstroke in type−B1 flapping mechanism wing 3D wing surfaces at different time steps; (**u**–**x**) upstroke in type−B1 flapping mechanism wing 3D wing surfaces at different time step.

**Figure 16:**
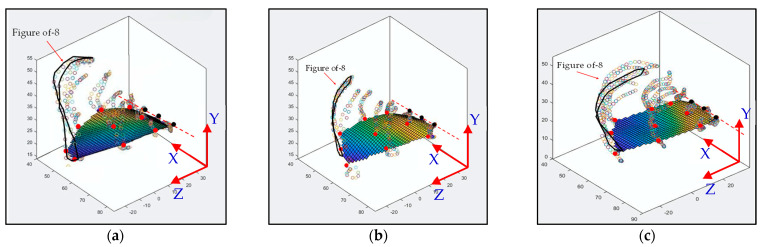
Figure-of-8: (**a**) type−A1 flapping mechanism; (**b**) type−B flapping mechanism; (**c**) type−B1 flapping mechanism.

**Figure 17:**
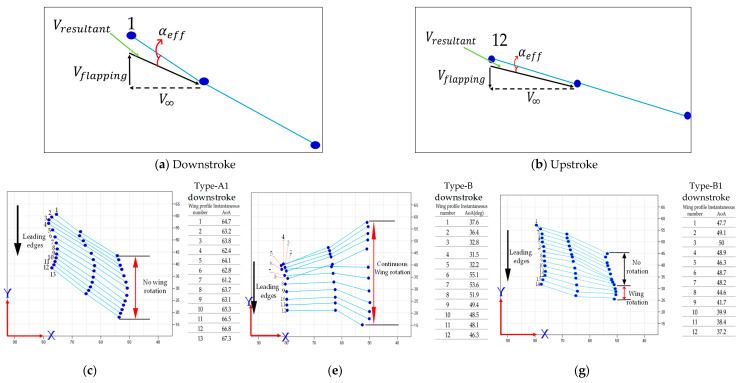

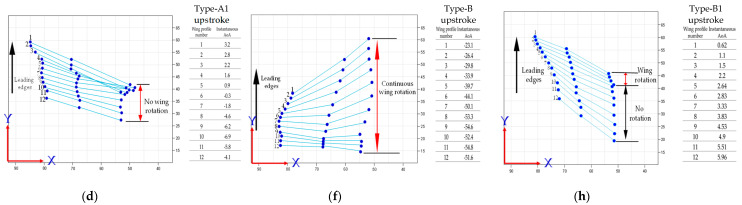
The 2D wing profile at mean aerodynamic chord: (**a**) AOA calculation in downstroke motion; (**b**) AOA calculation in upstroke motion; (**c**) type−A1 flapping mechanism’s downstroke; (**d**) type−A1 flapping mechanism’s upstroke; (**e**) type−B flapping mechanism’s downstroke; (**f**) type−B flapping mechanism’s upstroke; (**g**) type−B1 flapping mechanism’s downstroke; (**h**) type−B1 flapping mechanism’s upstroke.

The type-B1 mechanism had both the translational flapping and selective leading-edge twisting with the total cruising lift of 84 gf obtained at the cruising condition of 35° inclined angle, 5V driving voltage, and 3.0 m/s wind speed. The authors used MATLAB software to interpolate the 12-point trajectory of Figure 14b to the 3D wing surfaces of Figure 15q–x to demonstrate the flapping feature. The downstroke motion is shown in Figure 15q–t and the upstroke is shown in Figure 15u–x, which has been linked to the lift signal in Figure 14a and the figure of-eight wing tip motion in Figure 16c. The 2D airfoil cuts of the 3D surfaces were also taken using MATLAB at the mean aerodynamic chord and are shown in Figure 17e,f. Combining these 2D airfoil cuts and the relative incident velocity at the leading edge [46], it is easy to understand the instantaneous lift change of the flapping motion with continuous leading-edge twisting.

On checking the lift peaks in Figure 14a, strong leading-edge twisting motion plays important roles in this type-B1 mechanism. Again, both the first peak during the downstroke and the center peak during the upstroke are related with wake capture, but the second strong peak during the downstroke or at the supination is definitely relevant to the leading-edge twisting. By cross checking 3D profiles of the type-B1 mechanism in Figure 15q–x and 2D cuts in Figure 17e,f, the inclined angle behavior restores the similar trend of the simple flapping of the type-A1 mechanism during the downstroke and upstroke and no negative instantaneous AOA is observed throughout the flapping cycle. Instantaneous AOA is shown in Figure 17e for the downstroke, and it varied from 37.2° to 50° and the maximum lift produced was 84 gf. During the upstroke in Figure 17f, the instantaneous AOA varied from 0.62° to 5.96° and the AOA always stayed positive, which resulted in more lift forces when averaging the overall lift data for one full flapping cycle.

In addition, the figure-of-eight in Figure 16c also restores the similar trend of the simple flapping or even gets better (almost like a spherical trajectory) and reveals a strong FSI effect due to the strong thrust force generation and results in a larger cruising speed and lift as a consequence.

All the three flapping mechanisms actuated translationally via servo motors while type-B and type-B1 had bevel gears for leading-edge twisting throughout flapping. R·Φ in Equation (1) represents the disc radius multiplied by the stroke angle for translational stroke distance, which is equal to r· Ψ represents the bevel gear radius multiplied by the leading-edge twisting angle, and Table 5 demonstrates the design values of variables R, Φ, r, and Ψ in the three mechanisms.

The condition of Equation (1) is always satisfied for the continuous leading-edge twisting of the type-B mechanism. This is evident from the type-B mechanism in Table 5, the leading-edge twisting angle Ψ should be designed as 162° by the assigned stroke angle Φ of 96°. Due to the non-rigid characteristics of the 3D-printed parts, the measured leading-edge twisting angle Ψ is degenerated to 144°. Similarly, for the case of the type-B1 mechanism, the continuous leading-edge twisting angle Ψ should be 192° by the assigned stroke angle Φ of 87°. (The reason why a stroke angle of 87° for type-B1 is smaller than the 96° for type-B may be due to the more complicated mechanism design under the same driving power.) After considering the duty percentage of rotation of 74.7% mentioned in the caption of Figure 12, the designed value of the leading-edge twisting angle Ψ is reduced to 143°. Moreover, due to the flexible material and tolerance reasons, the measured leading-edge twisting angle Ψ is greatly degenerated to 108°.

In the following section, more explanation about the different types of mechanisms will be addressed in terms of 3D trajectory analyzed by the stereophotography and Kwon3D/KwonCC software.

Here, in Figure 17a,b, the graphical representation of the resultant velocity and instantaneous AOA is demonstrated. The same method is followed for the other mechanisms to identify the instantaneous AOA.

The authors summarized the pros and cons of all three flapping mechanisms in Table 6 and found that the type-B1 mechanism with adjustable mechanical stoppers has better performance than the other two flapping mechanisms.

Table 7 shows the translational flapping stroke angle Φ and leading-edge twisting angle Ψ obtained from the Kwon3D analysis. The 2D cut section at the mean aerodynamic chord from Figure 17 revealed the leading-edge twisting angle of all three flapping mechanisms and the angles were measured. Measured translational flapping stroke angle Φ and leading-edge twisting angle Ψ angle were adopted from Table 5 for comparison. The angle variation (3.7–7.3%) may be due to the computation error (1.0–1.5%) of KwonCC, and calibration errors and Kwon3D software and other manual manipulation errors during the experiments.

### 3.4. Flapping Trajectory of Rigid-Body Model Fitting to the Kwon3D/MATLAB Visual Motion Data

In order to simplify the comparison and verify the 2D cut section from MATLAB, the authors additionally used the theoretical model of rigid body flapping trajectory to obtain the position of the wing profiles for all three mechanisms. They are in terms of precisely given functions with respect to time, following what has been popularly adopted in some prior user-defined functions (UDF) of CFD simulation works [46,47,48]. The first important time-changing variable is the instantaneous AOA $\left(\alpha t\right)$:

(2) $$\alpha (t)=-\frac{\Psi }{2}+\frac{\Psi }{2}sin(\omega t+\gamma )$$

where t is time; Ψ is the leading-edge twisting angle; γ is the phase angle, and to be zero, the servo motors were set as symmetrical wing rotation at the leading edge; ω is the flapping frequency. The second time-changing variable is the flapping amplitude A(t) of the center of mass as shown in Equation (3).

(3) $$A\left(t\right)=\frac{A_{0}}{2}\left[\cos\left(\omega t\right)+1\right]$$

where A_0_ is the maximum amplitude of the flapping motion and is equal to the quarter span b multiplied with the stroke angle Φ.

**Figure 18:**
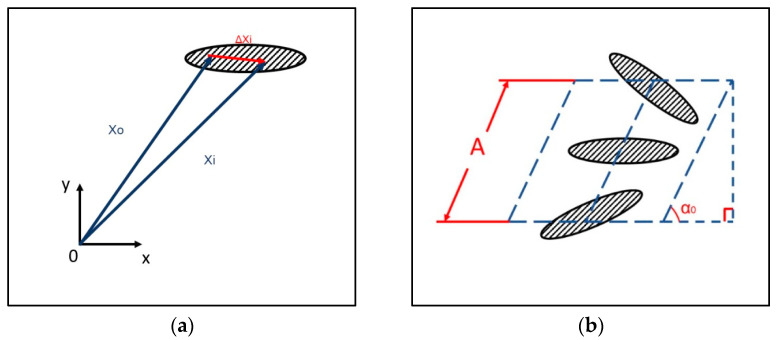
(**a**) Relative coordinate sign of a 2D wing; (**b**) displacement of the centroid.

Mass center of the wing section (x_0_, y_0_) is described by the flapping amplitude Equation (3) projected along x and y coordinates as Equation (4).

(4) $$\left\{\begin{array}{c} x_{0}\\ y_{0} \end{array}\right\}=A\left(t\right)\left\{\begin{array}{c} cos\;\alpha_{0}\\ \sin\alpha_{0} \end{array}\right\}$$

where $\alpha_{0}$ is the flapping plane angle of the flapping wing (almost vertical herein). Figure 18 shows the 2D schematic of any boundary point of a flapping airfoil which comprises the mass center translation and the rigid body rotation with respect to the mass center as below:

(5) $${\tilde{X}}_{i}=\left\{\begin{array}{c} x_{i}\\ y_{i} \end{array}\right\}={\left\{\begin{array}{c} x_{0}\\ y_{0} \end{array}\right\}}_{mass\_center}+\left[\begin{array}{cc} \cos\alpha \left(t\right) & -\sin\alpha \left(t\right)\\ \sin\alpha \left(t\right) & \cos\alpha \left(t\right) \end{array}\right]{\left\{\begin{array}{c} ∆x_{i}\\ ∆y_{i} \end{array}\right\}}_{rigid\_body\_rotation}$$

The points of the wing airfoil other than the mass center are, with the displacement (Δx_i_, Δy_i_), relative to the mass center at time zero and rigidly rotate with respect to the instantaneous AOA in Equation (2). The instantaneous AOA is already found from the MATLAB 2D cut section and shown in Figure 17 and Table 7, which eases the boundary condition input for the following calculations.

**Figure 19:**
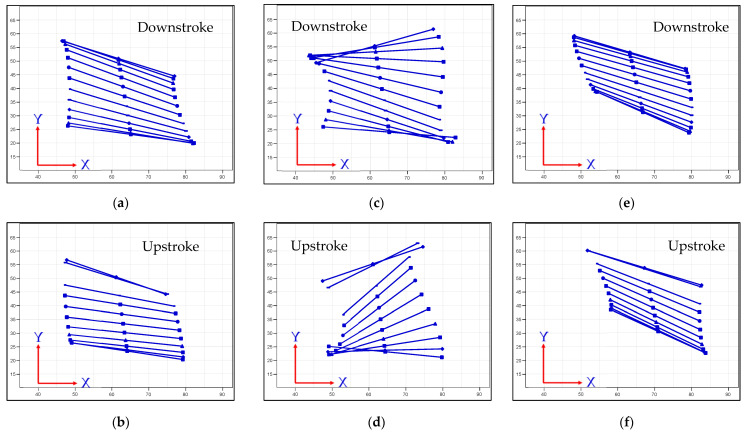
Rigid-body wing profiles of flapping wing mechanisms: (**a**,**b**) type-A1: normal servo mechanism (twist = −25°; phase = 40°; stroke = 72°; plane = 100°; inclined = 25°); (**c**,**d**) type-B: servo-bevel gear mechanism(twist= −90°; phase= −25°; stroke = 92°; plane = 90°; inclined = 35°); (**e**,**f**) type-B1: servo-bevel gear mechanism with adjustable mechanical stopper (twist = 15°; phase = 50°; stroke = 81°; plane = 95°; inclined = 35°).

From Table 7, the measured angle from Kwon3D software revealed that the maximum stroke angles Φ are 72°, 92°, and 81° for the Type-A1, -B, and -B1 mechanisms, respectively. The twist angles Ψ are taken as 0°, 134°, and 102° for the Type-A1, -B, and -B1 mechanisms, respectively. The inclined angles are 25°, 35°, and 35° for the Type-A1, -B, and -B1 mechanisms, respectively. By substituting all these parameters into Equations (2)–(5), the rigid-body wing profiles for the three proposed flapping mechanisms in this work were found and shown in Figure 19.

From Figure 19, it is also inferred that the wing profiles in the type-B1 mechanism are always in positive AOAs and the lift forces generated in this mechanism are always positive, too. The other two flapping mechanisms have some points of negative AOAs which result in negative lift generation. This observation is similar to the results of the real flexible wing cases in Figure 17 and Table 7.

The effect discussion of the instantaneous AOA values of Figure 17 and Figure 19 on the final lift performance herein is based on the quasi-steady observation and may not exactly match with the absolute lift values of real cases of unsteady flapping flights (Figure 8a, Figure 11a, and Figure 14a) [48]. More detailed CFD may be necessary in the future for further comparison of the unsteady aerodynamic forces. The simplified model of Equations (2)–(5) with the parameter values of stroke angles Φ and twisting angle Ψ deduced from the visual motion experiment in this work will be very beneficial to building the UDFs of the unsteady boundaries for the flapping wing models in future CFD studies [10]. From the time-varying AOA obtained from stereo visual sensing, the authors can identify the numerical values of the parameters in the theoretical trajectory model. We try to fit the AOA time history to define the parameters for the figure-of-eight trajectory.

## 4. Conclusions

In this work, three flapping mechanisms were designed and fabricated using rapid 3D printing technology and named as (1) flapping motion only (type-A1), (2) flapping with continuous leading-edge twisting (type-B), and (3) flapping with restricted leading-edge twisting via mechanical stoppers (type-B1). These mechanisms were tested under a low-speed wind tunnel to enumerate the aerodynamic forces, and the cruising conditions were determined.

The type-B1 flapping mechanism exhibited 32.9% extra lift compared to the type-A1 flapping mechanism (close to 35% extra impact on producing lift due to leading-edge twisting) and 64% more lift than the type-B mechanism. The type-B flapping mechanism of continuous leading-edge twisting mechanism was not appropriate to produce large lift due to there being too much wing rotation which led to a lower average positive lift and also an inability to produce zero net thrust.

To support the wind tunnel results, a visual motion experiment was carried out under a 1m^3^ calibration frame and the wing flapping motion was obtained by the stereo photography. Kwon3D software was incorporated to achieve the 3D coordinates of all markers on the flapping wing. Both translational and leading-edge twisting angles were measured using Kwon3D analysis. MATLAB software was furthermore employed to create the 3D fitted surfaces so as to explain the leading-edge twisting and the characteristics of lift signal peaks at the same time.

From the 3D surfaces of all three flapping mechanisms, the leading-edge twisting was observed in the type-B and type-B1 mechanisms and no leading-edge twisting in type-A1. However, the type-B continuous leading-edge twisting was outpacing the demand and generated negative net thrust. While controlling the leading-edge twisting using mechanical stoppers in the type-B1 mechanism, it was noted that the lift was increased.

Furthermore, the 2D cut section from MATLAB at the mean aerodynamic chord facilitated an understanding of the airfoil profile at each time frame, and the leading-edge twisting timing and the instantaneous AOA were found. The 2D profiles in the type-B1 mechanism revealed that the instantaneous AOA remained positive throughout the flapping cycle and was able to produce more lift than the other two mechanisms.

A theoretical model was also derived to obtain the wing profiles of all three flapping mechanisms by inputting parameter values, such as stroke angle Φ, twisting angle Ψ, and so on, inferred from the visual motion experiment to compare with the 2D cut section profiles from MATLAB.

In the current study, the 3D surfaces and 2D cut section profiles from MATLAB were based on a quasi-steady analysis. In future, a more comprehensive CFD analysis needs to be carried out to verify and compare the unsteady aerodynamic forces of flapping wings through the UDFs modeled by wing profiles generated by the visual motion experiments in this work.

## 5. Patents

“Leading-edge twisting structure of flapping wing micro air vehicle” with Taiwan patent number I739354.

## Figures and Tables

**Table 1:** Dimensions and details of wind tunnel.

Parameters	Metric
Length	15 m
Width	2.2 m
Height	1.8 m
Wind speed range	1–28 m/s
Lowest turbulence intensity	0.5%

**Table 2:** Cruising conditions of type-A1 flapping mechanism.

Inclined Angle	Flapping Frequency	Driving Voltage	Cruising Speed	Cruising Lift
10°	2.5 Hz	5V	4.0 m/s	26 gf
15°	2.5 Hz	5V	4.0 m/s	49 gf
20°	2.5 Hz	5V	3.0 m/s	45 gf
**25°**	**2.5 Hz**	**5V**	**3.0 m/s**	**63.2 gf**
35°	2.5 Hz	5V	1.7 m/s	35 gf

**Table 3:** Cruising conditions of type-B flapping mechanism.

Inclined Angle	Flapping Frequency	Driving Voltage	Cruising Speed	Cruising Lift
10°	2.5 Hz	5V	3.0 m/s	28 gf
15°	2.5 Hz	5V	2.5 m/s	40.3 gf
20°	2.5 Hz	5V	2.2 m/s	47 gf
25°	2.5 Hz	5V	1.6 m/s	45.5 gf
**35°**	**2.5 Hz**	**5V**	**1.5 m/s**	**51.1 gf**

**Table 4:** Cruising conditions of type-B1 flapping mechanism.

Inclined Angle	Flapping Frequency	Driving Voltage	Cruising Speed	Cruising Lift
10°	2.5 Hz	5V	3.6 m/s	34 gf
15°	2.5 Hz	5V	3.8 m/s	57 gf
20°	2.5 Hz	5V	3.25 m/s	64 gf
25°	2.5 Hz	5V	3.1 m/s	66 gf
**35°**	**2.5 Hz**	**5V**	**3.0 m/s**	**84 gf**

**Table 5:** Stroke angle and leading-edge twisting angle in all three flapping mechanisms of this work.

Mechanism Type	R (mm)	Φ (deg) Stroke Angle	r (mm)	Ψ (deg) Leading-Edge Twisting Angle	
				Design	Measured
Type-A1: Servo mechanism	14.6	75	Nil	Nil	Nil
Type-B: Servo + bevel gear	14.6	96	8.63	162	144
Type-B1: Servo + bevel gear + stopper	14.6	87	6.62	143	108

**Table 6:** Pros and cons of the three mechanisms studied in this work.

Mechanism Type	Wake Capture		2nd Peak Due to Delayed Stall	FSI Effect/ Figure-of-8	Negative AOA at Stroke Reversal
	1st Peak	3rd Peak			
Type-A1: Servo mechanism	Medium	Medium	Medium	Medium	None
Type-B: Servo + bevel gear	Medium	Weak	Weak	Weak	Yes
Type-B1: Servo + bevel gear + stopper	Strong	Strong	Strong	Strong	None

**Table 7:** Stroke angle and leading-edge twisting angle in flapping mechanisms compared with the Kwon3D results.

Mechanism Type	Measured Stroke Angle Φ (deg)	Stroke Angle by Kwon3D Φ (deg)	Measured Leading-Edge Twisting Angle Ψ(deg)	Leading-Edge Twisting Angle by Kwon3D Ψ(deg)
Type-A1: Servo mechanism	75	71.5	Nil	Nil
Type-B: Servo + bevel gear	96	92.4 (96.3%)	144	134.3 (93.3%)
Type-B1: Servo + bevel gear + stopper	87	80.6 (92.6%)	108	101.8 (94.3%)

## Data Availability

Not applicable.
